# Evaluation of the Antibacterial Activity of *Mentha Longifolia* Essential Oil against *Enterococcus faecalis* and its Chemical Composition 

**DOI:** 10.30476/dentjods.2025.101488.2304

**Published:** 2025-09-01

**Authors:** Mohammad Ghazizadeh, Abolfazl Davoodabadi, Sohrab Kazemi, Azadeh Harandi, Maryam Ghasempour

**Affiliations:** 1 Student Research Committee, Babol University of Medical Sciences, Babol, Iran.; 2 Infectious Diseases and Tropical Medicine Research Center, Babol University of Medical Sciences, Babol, Iran.; 3 Cellular & Molecular Biology Research Center, Health Research Institute, Babol University of Medical Sciences, Babol, Iran.; 4 Dental Material Research Center, Institute of Health, Babol University of Medical Sciences, Babol, Iran.; 5 Oral Health Research Center, Institute of Health, Babol University of Medical Sciences, Babol, Iran.

**Keywords:** *Enterococcus faecalis*, Mentha, Root canal

## Abstract

**Background:**

*Enterococcus faecalis* (*E. faecalis*) is a multidrug resistant pathogen that can cause persistent infections within the root canal system, which poses major challenges in dentistry and is involved in the majority of endodontic failures.

**Purpose:**

This investigation was conducted to evaluate the antibacterial properties of *Mentha Longifolia* (M. longifolia) essential oil as a root canal irrigant against *E. faecalis* and its compounds.

**Materials and Method:**

In this *in vitro* study, 60 primary canines were divided into five groups: G1: Normal saline (negative control), G2: Normal saline, G3: 2.5% sodium hypochlorite (NaOCl), G4: 2% Chlorhexidine (CHX), and G5: M. longifolia essential oil based on minimum inhibitory concentration (MIC). The standard strain suspension of *E. faecalis* (ATCC 29212) was injected into the canal of all groups, except for the negative control group. After four weeks, the groups were washed with 2 ml of the introduced solutions and sampled by paper point to compare the antibacterial effect of these solutions. Broth microdilution method was used to determine the MIC, while Gas chromatography-mass spectrometry (GC/MS) was used to evaluate the chemical compositions of the essential oil. Data were analyzed based on one-way analysis of variance (ANOVA). Then, pairwise comparisons of groups were performed using Tukey's test.

**Results:**

The MIC of M. longifolia against *E. faecalis* was 10%. As a root canal irrigant, its highest antibacterial impact was associated with 2.5% NaOCl, 2% CHX, 10% essential oil, and normal saline. The difference was not statistically significant in antibacterial effect between 2.5% NaOCl and 2% CHX (p Value=0.64). However, the difference was statistically significant in antibacterial effect between 2.5% NaOCl and 10% M. longifolia, 2.5% NaOCl and normal saline, 2% CHX and 10% M. longifolia, 2% CHX and normal saline, and 10% M. longifolia and normal saline (*p*< 0.001). Nineteen compounds were identified in the chemical analysis of M. longifolia, among which Piperitenone oxide (64.68%) and Piperitone oxide (23.68%) were the major compounds.

**Conclusion:**

M. longifolia essential oil (10%) was effective against *E. faecalis*. The highest antibacterial effect of root canal irrigants was observed in 2.5% NaOCl, 2% CHX, 10% essential oil, and normal saline, respectively.

## Introduction

Pulp and periapical diseases are primarily caused by bacteria. Based on its properties, the *Enterococcus faecalis* (*E. faecalis*) bacterium is categorized as a Gram positive, facultative anaerobic microorganism. The ability of this bacterium to attack dentin tubules and resistance to difficult conditions of the root canal has placed it among permanent and chronic endodontic microorganisms [ 1 ]. Efficacious debridement and proper disinfection of the root canal are required to achieve long-lasting root canal treatment [ 2 ]. A good and efficient root canal irrigant should kill a wide range of bacteria, break down any leftover dead tissue, neutralize toxins, and remove the smear layer [ 2 - 3 ]. An example of a proper irrigant engaged in root canal treatment is sodium hypochlorite (NaOCl), which is potent enough to show antibacterial properties against a wide range of bacteria and can attain properties to disintegrate necrotic pulp tissue. However, NaOCl has some intrinsic disadvantages [ 4 ]. It causes periapical tissue toxicity, is unable to eliminate the smear layer totally, brings about changes in the physical structure of the root canal walls, and has an unfavorable smell and taste [ 4 ]. On the other hand, 2% chlorhexidine (CHX), a cationic irrigant, is appropriately suitable for periapical tissues [ 5 ]. Although CHX possesses a wide range of antibacterial properties, it is unable to dissolve necrotic tissue . When CHX is utilized as an irrigant for a long period, it comes with various side effects that restrict its use. These side effects include discoloration of teeth, ageusia, oral mucosa irritation, dry mouth, and discoloration of the tongue . A flowering plant from the Lamiaceae family named *Mentha Longifolia* (*M. longifolia*) is a perennial plant that grows in the tropical regions of Central and Southern Europe, Southwest Asia, and North Africa. It has approved therapeutic properties in the treatment of gastrointestinal disorders, emesis, eating disorders, ulcerative colitis, and hepatic diseases [ 11 - 12 ]. The bactericidal and antioxidant properties of this plant have been evaluated in both forms of essential oil and extract [ 13 - 14 ]. Gram-positive bacteria seem to be more vulnerable to the essential oil of this plant compared to Gram-negative bacteria [ 15 ].

So far, no in vivo or *in vitro* study has been performed about the effect of *M. longifolia* essential oil (which also grows in Northern Iran) against *E. faecalis*. Therefore, the present *in vitro* study was conducted to investigate the antibacterial effect of *M. longifolia* essential oil as a root canal irrigant against *E. faecalis* and its compounds. 

## Materials and Method

This *in vitro* study was approved (approval code: IR.M-UBABOL.HRI.REC.1398.168) by the Ethics Committee of Babol University of Medical Sciences. *M. longifolia* is a wild plant and it was collected from the countryside of Qaemshahr, Northern Iran. It was also approved by the researchers of the Botany Department of Jihad Agricultural Science and Natural Resources Research Center of Mazandaran province. First, the aerial parts of fresh spring *M. longifolia* were crushed, and the essential oil was then extracted using water distillation and Clevenger apparatus (Schottduran-Germany). The *E. faecalis* ATCC (29212) standard strain was collected from the microbial bank of the Department of Microbiology, Babol University of Medical Sciences. 

To determine the minimum inhibitory concentration (MIC) of *M. longifolia* essential oil, micro-broth dilution method was utilized in 96-well plates according to the recommendations of the National Committee for Clinical Laboratory Standards (NCCLS) [ 16 ]. For this purpose, 40μl of Brain Heart Infusion Broth (BHI Broth) culture medium (Biolife-Italy) containing 5% dimethyl sulfoxide (DMSO) (Scharlau-Spain) was added to the first well, and 100μl of culture medium, with 5% DMSO used as essential oil solvent, was added to the next wells of the microplate. Then, 160μl of the essential oil was added to the first well and was mixed with the medium and 100μl was removed and added to the second well. The essential oil of the plant was diluted 1:2 at each stage. In the next step, all wells except the last well in each row (negative control) were added with 1μl of bacterial suspension at a concentration of 0.5 McFarland. While keeping the temperature at 37°C, incubation was done for 24 hours. Consequently, MIC was determined as the last well (concentration) that showed no growth, or turbidity. The test was repeated three times to determine a definitive MIC.

Sixty extracted primary canines without fracture, abnormal curvature, and pathological resorption were selected and soft tissue remnants were cleaned by a periodontal curette. Subsequently, the teeth were kept in 0.5% NaOCl solution (Nik Darman Asia-Iran) for 24 hours and then restored in 0.9% normal saline at an ambient temperature throughout the experiment. In addition, normal saline was replaced every week. Samples were decoronated using a bur with water and roots of 10 mm length which finally remained. Within root canals, a #15 K-file (Mani-Japan) was introduced and speared throughout the apex. Then, the teeth were filed up to #35 K-file with a length of about 1mm shorter than working length. Finally, irrigation of the teeth was performed with 3ml of 17% ethylenediaminetetraacetic acid (EDTA) solution (Morvabon-Iran) and 5.25% Na-OCl. Each solution remained in the canal for ten minutes to achieve the elimination of the smear layer [ 17 ]. The apex of the root canal was closed by using a composite (DenFil-South Korea) and the roots were covered by nail polish. Each root was firmly secured in the microtube with putty (Coltene-Switzerland) in a way that the root was placed vertically in the microtube. Teeth were divided into five groups of twelve, and every single group was assigned to a box. For sterilization, they were placed in an autoclave (Reyhan Teb-Iran) at 121°C for 30 minutes. 

First, by keeping the temperature at 37°C, the standard strain of *E. faecalis* was cultured on a blood agar medium for 48hours. Then, a microbial suspension with a concentration of 0.5 McFarland was prepared in a sterile saline. Afterward, 100μl of bacterial suspension was added to the root canals of all groups except the negative control group by an insulin syringe [ 18 ]. After this step, the canals were filled with BHI Broth culture medium, and *E. faecalis* was allowed to grow for four weeks. The culture medium was renewed every 48 hours. To confirm the lack of growth of another microorganism, Gram staining, catalase and Bile Esculin tests were performed. In the next step, to confirm bacterial colonization inside the root canal, two samples from each group were collected using a #35 sterile paper point (Meta, South Korea).

The study groups were categorized into the following groups; Group 1: Normal saline (Negative control) without bacteria ( in other groups, we cultured the canals with *E. faecalis* and irrigated them with the introduced substances), Group 2: Normal saline, Group 3: 2.5% NaOCl, Group 4: 2% CHX, and Group 5: *M. longifolia* essential oil solution based on MIC.

**Figure 1:**
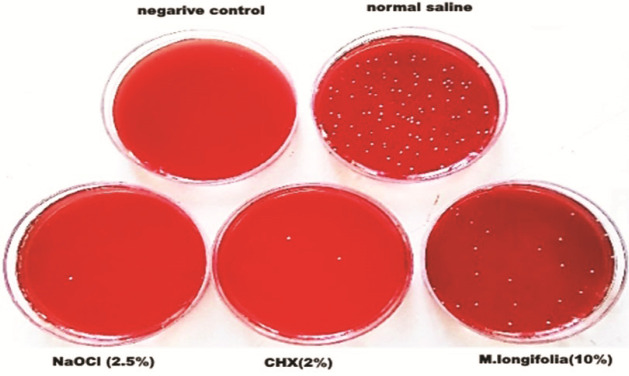
Samples of colonies grown after root canal irrigation in the studied groups on blood agar medium

Irrigation of the groups has been done with 2 ml of the introduced solutions and finally, irrigation of all groups has been done with 3 ml of normal saline. The sterile paper point #35 was positioned within the root canal for one minute and subsequently, the paper point was placed in the microtube which contained 450 ml of normal saline. The homogenization of solution was done for 60 seconds using a vortex (Labnet-Iran). Following the dilution process, the 100μl of the diluted solution was shifted to a blood agar medium by a sampler and was then cultured. The incubation of the plates was done at 37°C for 48 hours and fully developed colonies were calculated (Figure 1) [ 18 ]. 

Essential oil composition analysis was performed by GC/MS (Agilent Technologies-USA) in the Pharmacology Laboratory of the Babol Medical School. Helium gas (with a purity of 99.999%) was injected into the column of a gas chromatography machine at a rate of 0.8 ml/min. The temperature of the column was raised from 40°C to 208°C at a rate of 5°C/min. Inhibition index (IR) for all compositions of *M. longifolia* essential oil was calculated by injecting syringes of n-alkanes pattern of detected compositions with authentic standard retention time and spectroscopic patterns, respectively. Phytochemical analysis of *M. longifolia* essential oil was performed using a G/C Agilent 5977 gas chromatography-mass spectrometer. Identification of the phytochemical compositions of *M. longifolia* essential oil was determined by comparing the results of the GC/MS analysis with the reference retention time and spectral mass data of the NIST9 and wiley7 databases [ 19 ].

Data were analyzed using Statistical Package for the Social Sciences (SPSS) version 24 (SPSS Inc., Chicago, Illinois, USA). Moreover, the data were evaluated by employing one-way analysis of variance (ANOVA). Then, the pairwise comparisons of groups were performed using Tukey’s test. The *p* Value <0.05 was considered statistically significant. 

## Results

The MIC of *M. longifolia* essential oil against *E. faecalis* was 10%. The antibacterial effect of three irrigant solutions of 2.5% NaOCl, 2% CHX, and 10% *M. longifolia* essential oil as well as normal saline against *E. faecalis* is shown in Table 1.

**Table 1:** Comparison of the Mean of the Log10 CFU/ml and Standard Deviation (±SD) of *E. faecalis* in groups after root canal irrigation

Groups	Number of samples	Mean±SD	*p* Value
2.5% Sodium hypochlorite	12	2.23±0.35a	<0.001
2% Chlorhexidine	12	2.42±0.33a	
10% *Mentha Longifolia* essential oil solution	12	3.62±0.38b	
Normal saline	12	4.38±0.50c	

* One-way analysis of variance

There is no statically significant difference between the values with the same superscript (*p*>0.05)

As shown in Table 1, the lowest mean of log10 CFU/ ml was related to 2.5% NaOCl (2.23) and the highest mean was related to normal saline (4.38). Furthermore, in pairwise comparisons of groups, the results of Tukey’s test showed that these changes in 2.5% NaOCl and 2% CHX were not statistically significant (*p*= 0.64). However, a statistically significant difference was found between 2.5% NaOCl and 10% *M. longifolia* essential oil, 2.5% NaOCl and normal saline, 2% CHX and 10% *M. longifolia* essential oil, 2% CHX and normal saline, and 10% *M. longifolia* essential oil and normal saline (*p*< 0.001). As root canal irrigant, the highest antibacterial effect was primarily associated with 2.5% NaOCl, followed by 2% CHX, then 10% essential oil and finally normal saline.

**Figure 2:**
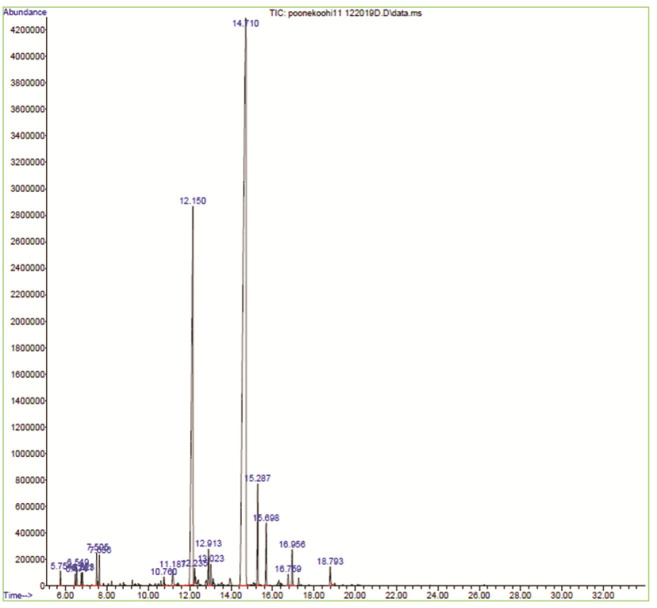
Gas chromatogram of the essential oil of *Mentha Longifolia* (L.) Hudson. The peak numbers correspond to the numbers of the compounds listed in Table 2

The composition of *M. longifolia* essential oil is shown in Table 2. In total, 19 compounds were identified with Piperitenone oxide (64.68%) and Piperitone oxide (23.68%) as major constituents. The gas chromatogram obtained from this *M. longifolia* essential oil is illustrated in Figure 2.

**Table 2:** Chemical compositions of *M. longifolia* essential oil by GC/MS

N	Compound	Retention Time (min)	Percent
1	Alpha Pinene	5.754	0.26
2	Sabinene	6.474	0.21
3	Beta Pinene	6.549	0.32
4	Beta-Myrcene	6.761	0.22
5	Amylethylcarbinol	6.823	0.26
6	Dl-Limonene	7.505	0.72
7	Cis-Ocimene	7.636	0.54
8	Carboxaldehyde	10.760	0.24
9	Furan	11.187	0.66
10	Piperitone oxide	12.150	23.68
11	Bicyclopentyl	12.235	0.29
12	Camphor	12.913	1.11
13	Diosphenol	13.023	0.54
14	Piperitenone oxide	14.710	64.68
15	Trans-Chrysanthemal	15.287	2.76
16	Trans-Caryophyllene	15.698	1.61
17	4-chloro-2,3-dimethyl	16.759	0.32
18	Germacrene-D	16.956	0.94
19	3-Chloro-4-t-butyl-6-methylpyridazine	18.793	0.66

## Discussion

Pulp and periapical diseases are primarily caused by bacteria and the severity of inflammation in these tissues is directly associated with the quantity of bacteria present within the root canal system. Therefore, successful endodontic treatment with a long-term prognosis is based on the cleansing of the root canal from bacteria and their products. *E. faecalis* is classified among bacteria involved in periapical lesions [ 1 ]. NaOCl is an increasingly used irrigant for root canal treatment and has high antimicrobial characteristics and has the ability to disintegrate dead tissues. However, one of the disadvantages of this irrigant is its toxicity when in contact with periapical tissue [ 4 ]. On the other hand, CHX as an irrigant has good antimicrobial activity and relatively little toxic effect; however, it does not have the ability to dissolve necrotic tissue [ 10 ]. The compatibility of 2% CHX irrigant solution with periapical tissues is due to its cationic properties [ 5 ].

Antimicrobial properties of plants have been studied in several fields [ 20 ]. In recent years, the advantages of medicinal herbs, including their naturalness and fewer undesirable effects, have attracted the attentions of many medical researchers. Moreover, the advantages of using plant essential oils as an alternative to industrial and chemical materials include their availability, lower toxicity, and cost-effectiveness [ 4 ]. One of the most important reasons for the antimicrobial effect of plant essential oils is their hydrophobic properties, which allows them to pass through the lipid membranes and mitochondria of bacteria, leading to their dysfunction. This disorder can lead to higher permeability of the membrane and changes in the osmotic pressure of the cell and the release of ions and other intracellular contents of bacteria, and eventually, the death of microorganisms [ 21 ].

Mechanical preparation and instrumentation have proven to be effective in reducing the number of bacteria in the canal, but this rate is only 50% because of the structural variations of root canal and the intricate canal anatomy (such as presence of lateral canals and narrow root canals) [ 22 ]. These anatomical variations and limitations are more noticeable in primary teeth [ 23 ]. Therefore, the use of chemical methods and root canal irrigants with antimicrobial properties that can overcome these anatomical limitations and if possible, eliminate microorganisms has been emphasized [ 24 ]. Considering differences between the root canal environment, clinical conditions, and the laboratory setting, the MIC of *M. longifolia* essential oil against *E. faecalis* was first determined using micro brush dilution in this study. This concentration of essential oil, used as a root canal irrigant, was then compared to the antibacterial effects of 2.5% NaOCl and 2% CHX.

Considering root canal irrigants in deciduous teeth, normal saline solution is usually employed in pulpectomy treatment in pediatric dentistry. The reduction in the bacterial count during endodontic treatments with this substance is only related to its flushing effect [ 25 ].

Our *in vitro* study showed that *M. longifolia* essential oil has antimicrobial effects against *E. faecalis* and its MIC is 10%. Stanisavljević *et al*. [ 26 ] in 2014 stated that the Serbian essential oil of *M. longifolia* (2%) had an inhibitory effect against *E. faecalis* and its diameter of growth inhibition zone was 17.5 mm, while the diameter of growth inhibition zone of the compared reference antibiotic (Ampicillin) was 16mm [ 26 ]. Moreover, Gulluce *et al*. [ 33 ] stated that *M. longifolia* essential oil has strong antimicrobial activity against all 30 microorganisms tested and MIC against *E. faecalis* was 62.50 µg/ml. This result may be explained by the high content of Cispiperitone epoxide (18.4%), pulegone (15.5%), and Piperitenone oxide (14.7%) in the essential oil of *M. longifolia* analyzed in their study. The results of a study by Abdel-Gwad *et al*. [ 34 ] showed that the major compounds of *M. longifolia* were Ementhone, Pulegone, Z-menthone, 1,8 Cineole, and Menthol. The inhibitory effect of this essential oil was higher against Gram-negative bacteria. Some reasons for the difference between the present study and other studies may include variations in the geographical area where the plants grow, the use of different parts of plants, and the methods of essential oil extraction. Another reason for the difference is the variation in the number of phytochemicals (the active ingredients of the medicinal plant) and antibacterial activity of *M. longifolia* essential oil [ 31 - 32 ].

Regarding as a root canal irrigant against *E. faecalis*, 10% *M. longifolia* essential oil was significantly weaker than 2.5% NaOCl and 2% CHX in this *in vitro* study and 10% *M. longifolia* essential oil was significantly stronger than normal saline. No study compared the antiseptic properties of *M. longifolia* essential oil with NaOCl and CHX against *E. faecalis*; therefore, no comparison could be drawn in this regard. In a study conducted by Santo *et al*. [ 27 ]the difference in the antibacterial effect of 2.5% NaOCl and 2% CHX against *E. faecalis* as a root canal irrigant was not statistically significant; this was similar to the results of our study.

Of note, 2.5% NaOCl and 2% CHX were used in this study, which showed higher antimicrobial activity against *E. faecalis* compared to *M. longifolia* essential oil. However, these two solutions are industrially refined products and it is used as a gold standard in research [ 28 ]. Separation and concentration of the active components of the plant may increase its antibacterial effect.

In this study, the results of analyzing *M. longifolia* essential oil compositions by GC/MS showed that the major components of *M. longifolia* essential oil are Piperitenone oxide (64.68%) and Piperitone oxide (23.68%). A study by Oumzil *et al*. [ 29 ] demonstrated that Piperitenone oxide and Piperitone oxide had antibacterial properties and Piperitenone oxide had a greater antibacterial effect compared to Piperitone oxide. Another study by Ghoulami *et al*. [ 30 ] demonstrated that the major constituents and phytochemicals (active ingredients of the medicinal plant) in essential oil prepared from *M. longifolia* grown in Morocco were Piperitenone oxide (25%) and Piperitone oxide (24%), which is consistent with the results of the current investigations regarding the type of primary compounds. According to the results of these studies [ 29 - 30 ], the most important components of *M. longifolia* essential oil include Piperitenone oxide, α-terpineol, Menthon, Menthofuran, 1,8-cineole, Cis-iso polygon, Cineole, cis-Piperitone epoxide, Eucalyptol, and Thymol. In an evaluation of *M. longifolia* from Garhwal Region of Western Himalaya, Ram S *et al*. [ 35 ] detected 55 constituents, which formed 97.5% of the total oil composition. The oil was characterized by a high number of oxygenated monoterpenes (74.0%) and sesquiterpene hydrocarbons (18.0%). The constituents of the oil included trans-Piperitone epoxide (48.7%), Piperitenone oxide (21.2%), germacrene D (9.8%), (E)-caryophyllene (2.3%), 2-hydroxy Piperitone (1.6%), α-humulene (1.5%), thymol (1.4%), and α-longipinene (1.0%). Multiple investigations have stated the main constituents of *M. longifolia* essential oil; however, there is a difference in the numerical amount of these compounds in *M. longifolia*. This difference in the number of constituents varies based on variables such as temperature, relative humidity, duration of sunshine, season, weather conditions, rainfall, and the use of different parts of the plant for extraction as well as the method adopted for extraction [ 31 - 32 ]. A limitation of this study is that M longifolia has different effects in different geographic environments. In this study, we only investigated its antibacterial effects in a specific area. Microbiological examination should be done by separating effective substances that have antibacterial properties. The antibacterial components of essential oil should be extracted and used in concentrated doses. Microbiological studies should be done using aforementioned substances. Considering the diversity of Iran's climate, more studies are recommended to find the greatest antibacterial effect of the plant.

## Conclusion

In this study, the MIC of *M. longifolia* essential oil against *E. faecalis* was 10%. The highest antimicrobial effect against *E. faecalis* as a root canal irrigant was related to 2.5% NaOCl, 2% CHX, 10% *M. longifolia* essential oil, and normal saline, respectively. Additionally, in the chemical analysis of the essential oil, Piperitenone oxide (64.68%) and Piperitone oxide (23.68%) were its major constituents. 
